# Comparing the Mechanical and Thermodynamic Definitions of Pressure in Ice Nucleation

**DOI:** 10.1021/acs.jpclett.5c03700

**Published:** 2026-02-12

**Authors:** P. Montero de Hijes, K. Shi, C. Vega, C. Dellago

**Affiliations:** † Faculty of Physics, 27258 University of Vienna, A-1090 Vienna, Austria; ‡ Department of Chemical and Biological Engineering, 12292 University at Buffalo, The State University of New York, Buffalo, New York 14260, United States; § Departamento de Química Física, Facultad de Ciencias Químicas, 16734 Universidad Complutense de Madrid, 28040 Madrid, Spain

## Abstract

Crystal nucleation studies using hard-sphere and Lennard-Jones models have shown that the actual (mechanical) pressure within the nucleus is lower than that in the surrounding liquid. Here, we use the mechanical route to obtain the pressure for an ice nucleus in supercooled water (TIP4P/Ice) at 1 bar and 247 K. From this pressure, we obtain the interfacial stress using a thermodynamic definition consistent with mechanical arguments. Moreover, we compare the mechanical pressure with the thermodynamic pressure of bulk ice at an equal chemical potential and the interfacial stress with the interfacial free energy. Furthermore, we investigate these properties on the basal plane. We find that unlike in hard-sphere and Lennard-Jones systems, mechanical and thermodynamic pressures agree for the nucleus, and the interfacial stress and free energy are comparable. However, the basal interface displays an interfacial stress nearly twice its interfacial free energy, suggesting that this agreement may be dependent on the system, underscoring the limitations of mechanical routes to solid–liquid interfacial free energies.

## Graphical abstract

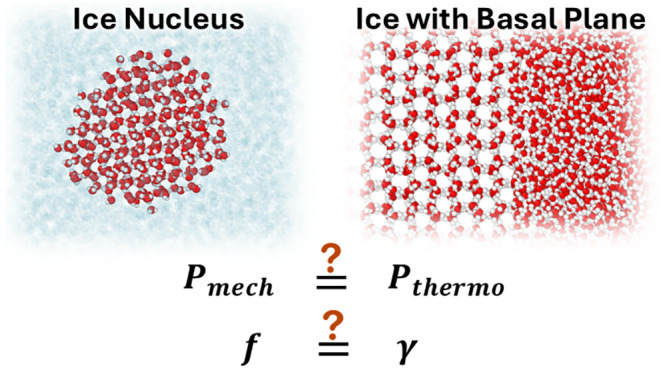

Ice nucleation is a critical process in nature and many technological fields.^1,2^ However, investigating critical nuclei is difficult, as nucleation events may take seconds to occur but may last for only a few nanoseconds. Computer simulations have significantly contributed to our understanding of this process.^3^ In particular, molecular dynamics simulations in the *NVT* ensemble provided a means not only to simulate crystal nucleation but also to stabilize critical nuclei,^4,5^ allowing us to study them in great detail. This has made it possible to carefully study the critical crystal nuclei of hard spheres (HS)^6,7^ and Lennard-Jones (LJ) particles,^8^ showing that the pressure inside is lower than that outside. Indeed, earlier work on hard spheres with short-range attractive interactions^9^ and on binary mixtures^10^ had implicitly indicated this, as reflected in the reported densities. These observations are, a priori, in contradiction with the Young–Laplace equation

$$\Delta p^{\mu }=\frac{2\gamma }{R}$$ 1

where Δ*p* ^μ^ is the difference in pressure assuming bulk phases (Δ*p* ^μ^ = *p*_I_h__^μ^ – *p*_w_ for ice Ih and water, respectively, in this work) and γ > 0, where γ is the interfacial free energy for a dividing surface defined as the surface of tension located at *R*. Following ref 6, *p*_I_h__^μ^ is the thermodynamic pressure of the ice nucleus (i.e., the pressure of bulk ice at the same chemical potential μ as the external fluid). The apparent conflict resolves once we recognize that the thermodynamic behavior of the crystal nuclei differs from that of the bulk crystal. Since eq 1 employs reference bulk states, it does not matter that at a given chemical potential and temperature, the actual (mechanical) pressure of the nucleus differs from that of the bulk.^6^ The true (mechanical) pressure of the nucleus is not directly related to γ; instead, it is related to interfacial stress *f*.^7^ Similar to the Young–Laplace equation, *f* can be defined thermodynamically as^7,11,12^

$$\Delta p=\frac{2f}{R}$$ 2

where Δ*p* = *p*_I_h__ – *p*_w_ is the actual difference in pressure between the two phases. Notice how true (mechanical) pressure *p*_I_h__ may be incorporated into a thermodynamic description (see also the Supporting Information). The origin of discrepancies in pressure between an ideal bulk crystal and the core of the nucleus at the same μ likely lies in the concentration of defects and possibly in the presence of strain.^7,12,13^

The radii of curvature *R* in both eqs 1 and 2 are often assumed to be equal for the sake of simplicity, but this is an approximation. Moreover, a mechanical route to γ in solid–liquid interfaces has not been successful to date,^3^ while several expressions have been proposed for *f*.^7,8,14,15^ At the root of all of this uncertainty is the arbitrariness in both (i) the location of the dividing surface in Gibbs’ thermodynamics and (ii) the definition of the pressure tensor in mechanics.^16^ Furthermore, the fact that γ = *f* in planar fluid–fluid interfaces and γ ∼ *f* in curved fluid–fluid interfaces (except for small droplets or confined systems where they may differ notably^17–26^ ) has often led to misunderstandings of the solid–liquid interface. Nevertheless, Gibbs already noted that the tension of the surface, meaning γ, did not refer to the true tension, meaning *f*. However, Gibbs wrongly believed that the differences would be negligible in most cases.^27^

Despite some remaining uncertainties, our understanding of solid–liquid interfaces has significantly improved thanks to computer simulations, which have allowed researchers to confirm that *f* is often negative in solid–liquid planar^28–33^ and spherical^3,6,8^ interfaces, drastically differing from γ, which is always a positive property. Moreover, systems may present *f* < 0 in some crystallographic planes while in others *f* > 0.^34,35^ Mechanically, *f* is rooted in the stress profile *S*(*r*) = *P*_N_(*r*) – *P*_T_(*r*), where *P*_N_(*r*) and *P*_T_(*r*) are the normal and tangential components, respectively, of the pressure tensor and *r* is the position along the axis normal to the interface. Notably, *S*(*r*) can be nonuniform with both positive and negative contributions depending on the interfacial layer.^36,37^ This nonuniformity was also observed at the water–vapor interface,^38^ even though in planar fluid interfaces *f* typically coincides with γ. Becker et al.^39^ suggested that the density difference between the two phases and the bonding energy play an important role in *f*, whereas Eriksson and Rusanov^40^ hypothesized that solid–liquid interfaces with high mobility could lead to *f* = γ. Recent work indicates that in a few cases, this could be the case for the planar ice–water interface. In ref 41, a thermodynamic formalism for fluid interfaces was reasonably successful in describing the planar ice–water interface using TIP4P/Ice.^42^ In contrast, studies using the mW model^43^ revealed in ref 33 showed that while *f* and γ are the same at one particular point of the coexistence line, they differ for most melting temperatures.

Here, we test whether the significant discrepancy between *f* and γ during nucleation observed in simple models also occurs for an important substance like water. In addition, we compare these two quantities for the basal plane at coexistence. In the following, we present the simulation details and then discuss the results—mainly γ, *f*, *p*_I_h__^μ^, and *p*_I_h__—for the ice nucleus. Finally, we examine the properties of the basal plane and conclude with a discussion and summary.

We use the TIP4P/Ice water model^42^ simulated using GROMACS-2021.3-mixed with (i) a time step of 2 fs, (ii) the Nosé-Hoover thermostat (relaxation time of 1 ps) fixed at 247 K (nucleus) and 270 K (basal plane), (iii) the Parrinello–Rahman barostat (relaxation time of 2 ps) fixed at 1 bar (isotropic for the nucleus and applied normal to the interface while the lateral dimensions of the box are kept fixed for the basal plane^44^), (iv) the particle-mesh-Ewald algorithm of order 4 and a Fourier spacing of 0.1 nm, (v) a cutoff of 0.9 nm in both Lennard-Jones and Coulomb interactions, and (vi) long-range corrections to the Lennard-Jones term. The dispersion correction contribution to the pressure yields −350.9 bar in the nucleus and −337.4 bar in the basal plane. Despite known artifacts,^45,46^ long-range cutoff corrections were retained to ensure consistency with the force-field parametrization, given the modest density difference between ice and liquid water.^42^

Following previous works on hard spheres and Lennard-Jones, we attempted to stabilize an ice nucleus in the *NVT* ensemble.^5–8^ However, after some failed attempts, we opted for generating trajectories in the *NpT* ensemble from a critical nucleus determined previously via the seeding approach.^47^ When the system contains a critical nucleus, it is in an unstable equilibrium. Although a small perturbation should cause the system to depart from its original equilibrium state, this is a stochastic process; there may exist trajectories in which the system does not quickly decide which final state it is going to take. The long-lived nucleus trajectories can therefore be used to analyze the mechanical properties in equilibrium in the *NpT* ensemble. We select the trajectories in which the nucleus size oscillates closely around the critical size for lifetimes at which, in most trajectories, the critical nucleus size would have already changed by ∼60*%*. We found six of 40 trajectories in which the nucleus survived for about 50 ns each at 247 K and 1 bar, allowing us to gather 300 ns. A limitation of the *NpT* versus *NVT* approach is that in *NpT* seeding we impose the critical nucleus structure, and this often removes relevant degrees of freedom that would have naturally evolved in a stable equilibrium, provided sufficient time was allowed, e.g., the stacking disorder of cubic and hexagonal ice.^48^ The system is shown in Figure 1 and contains 78 856 molecules, of which ∼1500 belong to the nucleus. For the planar interface, the system contained 20 736 molecules, about half being ice and half being liquid. The cross-sectional area defining the interface is determined to provide bulk behavior on the ice far from the interface.

**Figure 1:**
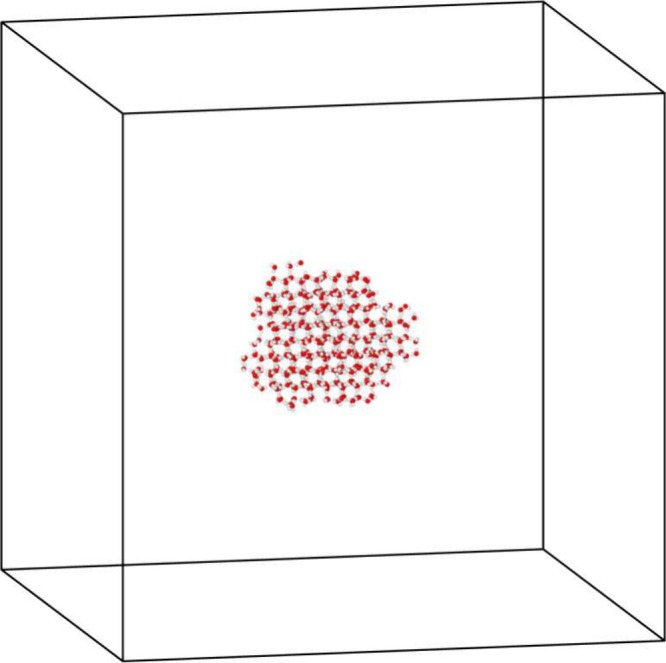
Snapshot of the critical nucleus configuration showing only the molecules belonging to the nucleus.

We proceed now by first describing the nucleus via the Young–Laplace equation (eq 1). Interfacial free energy γ has been widely investigated for the TIP4P/Ice water model.^41,49–51^ Therefore, we estimate γ from the supercooling Δ*T* using the empirical fit suggested in ref 41 γ = 26.6 – 0.174 × Δ*T* (mJ/m^2^), where Δ*T* = *T*_m_ – *T*. Given that melting temperature *T*_m_ of the model is 270 K, in this case γ ∼ 23 mJ/m^2^. Then, using eq 1, we can obtain pressure *p*_I_h__^μ^ of bulk ice at the same chemical potential as that of the external liquid, which is homogeneous throughout the whole system. To do so, we need first both water pressure *p*_w_, which is equal to 1 bar, i.e., the average pressure in the system (see the Supporting Information of ref 6), and the radius of the nucleus (*R*). In the introduction, curvature effects in γ due to the location of the dividing surface were not described for the sake of simplicity. Nevertheless, to be precise, we should denote γ and *R* in eq 1 as γ_s_ and *R*_s_, indicating that they are defined at a particular location of the dividing surface within the interfacial region. In particular, *R*_s_ is the surface of tension, at which the (notational) derivative of γ with respect to the arbitrary location of the dividing surface vanishes^52,53^ (see the Supporting Information for further details). Determining *R*_s_ requires free energy calculations to find the minimum in γ­[*R*], where the square brackets imply a variation with the location of the dividing surface and not with the actual radius. However, it has been shown empirically that a particular criterion based on order parameter $\overset{®}{q_{6}\;}$ ^54^ to classify ice-like and water-like molecules allows us to obtain a radius leading to agreement in free energy barriers with those obtained from free energy calculations.^47^ We refer to refs 41, 50, and 55 for details on this criterion. The number of ice-like molecules (*N*_I_h__) is estimated from $\overset{®}{q_{6}\;}$ using a threshold of 0.365 and a cutoff of 3.5 Å. Cluster radius *R*_s_ is then obtained from

$$R_{s}={\left(\frac{3N_{I_{h}}}{4\pi \rho_{I_{h}}^{\mu }}\right)}^{1/3}$$ 3

where ρ_I_h__^μ^ is the number density of bulk ice (i.e., the number of water molecules per unit of volume of ice I_h_ for TIP4P/Ice at 247 K and *p*_I_h__^μ^). Since the density of ice barely changes with pressure (∼0.5% over 500 bar^50,56^ ), we take it from bulk ice at 247 K and 1 bar even though bulk ice under such conditions will not have the same μ as the liquid but a lower value. The error introduced by this approximation is certainly smaller than that introduced in *R*_s_ via an empirical definition. The mass density of bulk ice at 247 K and 1 bar is ∼0.91 g/cm^3^. Hence, number density ρ_I_h__^μ^ ∼ 30.5 nm^–3^. As shown in Figure 2, *N*_I_h__ ∼ 1500 molecules and, finally, *R*_s_ is estimated from eq 3 to be ∼ 2.3 nm. From these estimates and using eq 1, we find that *p*_I_h__^μ^ ∼ 200 bar. This way of defining the nucleus pressure is termed thermodynamic pressure in the context of hard spheres in ref 6, where it is shown that such a pressure can be obtained directly by doing thermodynamic integration from coexistence. Note that the thermodynamic pressure is always larger than the external one. How far is this representation from the true mechanical pressure of the nucleus? How does γ compare to *f*? Gibbs believed that they would not be too different. However, in simple systems like hard spheres^6,7^ and Lennard-Jones,^8^ this is far from true since it has been observed that the true mechanical pressure is smaller than the external pressure, leading to *f* < 0, even though γ > 0 by definition.

**Figure 2:**
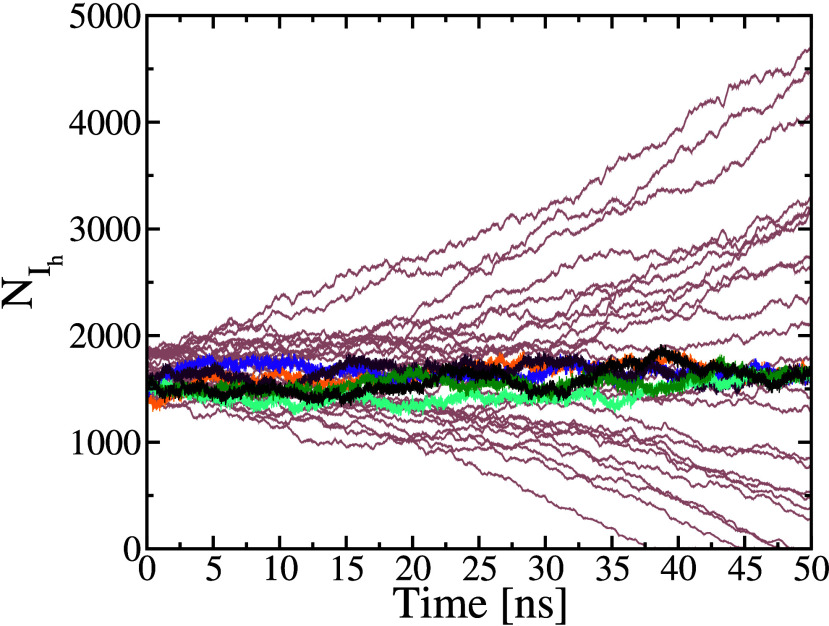
Time evolution of the number of ice-like molecules, *N*_I_h__, for the system in Figure 1. Brown lines show trajectories where the nucleus transited too early to acquire data, whereas the other colors show the trajectories that were finally employed for our analysis.

To address these questions for the ice–water interface, we analyze the trajectories to extract microscopic information. At each time step, the center of mass (COM) of the nucleus is determined. Molecules are then binned into concentric spherical shells extending from the COM of the nucleus toward the boundaries of the simulation box according to their distance from the COM. The local density at a given distance is obtained by counting the number of water molecules within each shell and dividing by the corresponding shell volume, yielding a density profile averaged over all directions. The resulting density profile is shown in Figure 3a. As one can see in this figure, the interface is not sharp. Only ice molecules within 2 nm of the COM are in a density plateau, whereas the interface spans almost the same length. Therefore, the actual radius of the nucleus is between 2 and 3.5 nm, suggesting that our previous estimate of *R*_s_ (∼2.3 nm) is reasonable. Also, the actual density is in agreement with the mass density of bulk ice, 0.91 g/cm^3^, within the uncertainty. Therefore, the true pressure of the nucleus (*p*_I_h__) is expected to be in agreement with that of *p*_I_h__^μ^.

**Figure 3:**
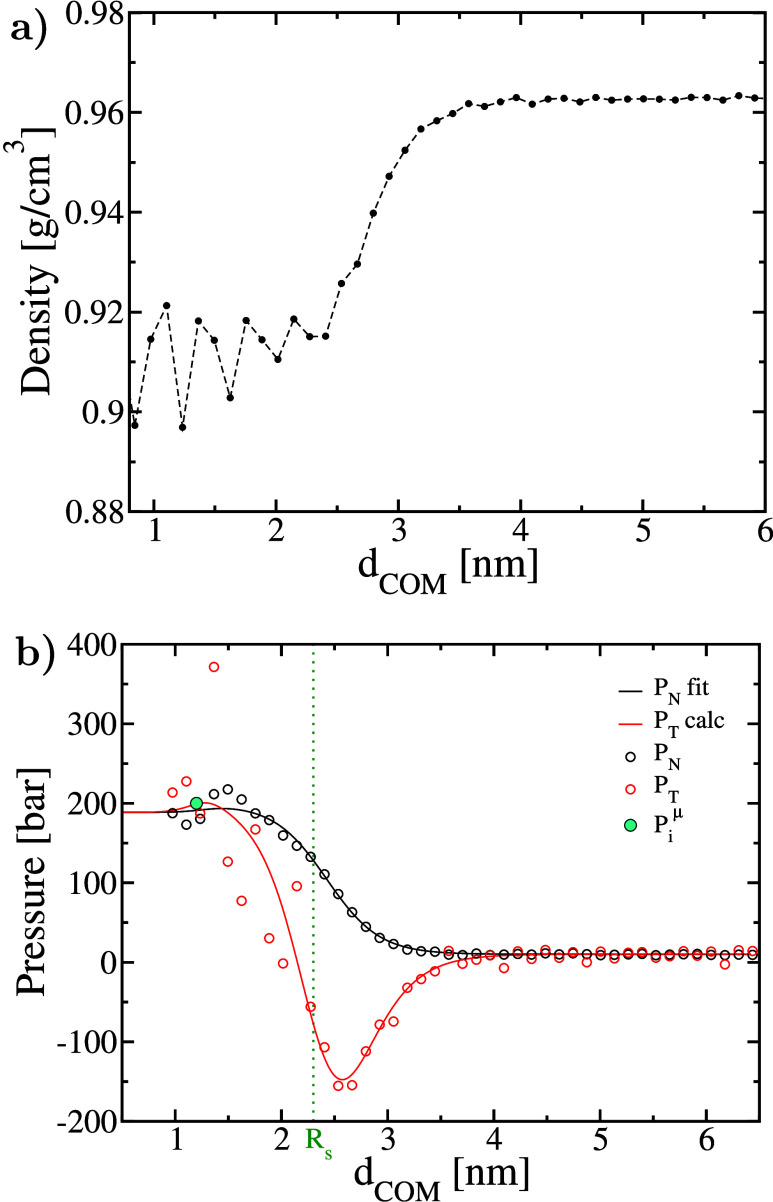
(a) Density and (b) pressure profiles as a function of distance *d*_COM_ from the center of mass of the ice nucleus. In panel b, the thermodynamic pressure (blue symbol) and position *R*_s_ of the surface of tension (vertical dotted green line) are also shown.

Indeed, *p*_I_h__ can be measured directly from molecular dynamics trajectories using the virial (or mechanical) route.^16^ Although the pressure tensor at a point is not uniquely defined, Shi et al.^57^ recently showed that it is possible to define a unique pressure tensor over a small region of space, roughly the range of the intermolecular forces, in a planar geometry. However, the validity of such a unique definition remains unclear in systems with curved interfaces. Here, we are interested in the local pressure of the nucleus far enough from the interface that the ambiguity in the pressure definition is of negligible order. In this work, we adopted the contour definition of Irving and Kirkwood,^14,58^ which is a straight line connecting two interacting molecules. All parameters in the pressure tensor calculations are consistent with those in the molecular dynamics simulations except for the Coulombic interactions. Incorporating the Ewald summation into the pressure tensor formulation is not trivial.^59^ Instead of implementing the Ewald summation directly, we adopted the shifted force version^60,61^ of the Wolf method^62^ to account for the long-range contribution to the local pressure tensor. This allows us to calculate the pressure tensor in a pairwise manner with accuracy comparable to that of the exact Ewald method. Derivation of molecular pressure tensor equations in spherical coordinates, calculation details, and source code are available in the Supporting Information.

In this system, the tensor has two non-zero components: normal (radial) pressure *P*_N_ and tangential pressure *P*_T_ (averaged from the polar and azimuthal pressures). As shown in Figure 3b, the pressure profile converges to the average pressure when it reaches the liquid far from the interface (within a 10 bar error). More importantly, it shows that the true pressure in the nucleus (*p*_I_h__) agrees with that of bulk ice at the same chemical potential as the system (*p*_I_h__^μ^); i.e., the true (mechanical) pressure inside the nucleus is ∼200 bar. The tangential component is rather noisy, so we estimated it, *P*_T,calc_, also from the fit to the normal component, *P*_N,fit_, combined with the hydrostatic equilibrium condition as explained in the Supporting Information. Interestingly, the interface is stretched even at negative pressures. However, we cannot quantify this effect with certainty due to the nonuniqueness in the definition of the local pressure tensor. In any case, the pressure profile is expected to cover significantly different pressures from about 200 bar at the core of the nucleus to negative pressure at the interface and again to standard pressure at the surrounding liquid.

With internal pressure *p*_I_h__ measured from the pressure profiles, we can then estimate *f* from eq 2 upon defining *R*. Just like with γ, the arbitrariness in the location of the dividing surface is expected to affect *f*. In the Supporting Information, we show that the *R* values in eqs 1 and 2 are not necessarily equal. The former, *R* = *R*_s_, is the surface of tension defined by Gibbs, whereas the latter, *R* = R*, is the true surface of tension. How to evaluate R* is, however, nontrivial, and no empirical rules have been suggested. Since the uncertainty in R* must be comparable to the interfacial thickness, we pragmatically estimate *f* for three different values, including *R*_s_ and the two limits of the interfacial region.

Considering the lowest (2 nm) and highest (3.5 nm) bounds for R*, we find that *f* should be between 20 and 35 mJ/m^2^. In particular, when R* ≈ *R*_s_, *f* ∼ γ ∼ 23 mJ/m^2^. Therefore, in the critical nucleus at standard pressure and 23 K of supercooling, *f* is comparable with γ for the TIP4P/Ice model. According to Eriksson and Rusanov,^40^ the high mobility of molecules at the ice–water interface would promote the equivalence, which is also supported by the lower density in the internal phase according to ref 39. Hence, the Gibbs assertion of their equivalence is not completely invalidated; rather, the extent to which they coincide seems to be dependent on the system.

Since the equivalence between *f* and γ seems to be contingent on the system, we now move from the nucleus to study a different case, the planar interface at coexistence for the basal plane (with Miller-Bravais indices 0001, Figure 4 a), whose interfacial free energy is well-known to be ∼27.2 mJ/m^2^.^41,63^ In this case, the temperature is 270 K instead of 247 K, and a single plane, instead of an average of planes, is exposed. As one can see in Figure 4b, the density profile exhibits an interface with a thickness similar to that of the nucleus (∼2 nm). The density profile is smoothed using a one-dimensional Gaussian filter with standard deviation σ = 3 Å.^64^ Moreover, in Figure 4c, we show the pressure profile across the basal plane for the normal and tangential components. Normal and tangential pressures, in this case, are perpendicular and parallel to the planar interface, respectively. They are calculated using the pressure tensor equations from ref 65 (see the Supporting Information). The normal pressure is constant at ∼7 bar within statistical uncertainties (standard deviation of ∼7.2 bar), confirming that the system has reached mechanical equilibrium. Only the diagonal elements in the pressure tensor are non-zero; all off-diagonal pressure components are confirmed to fluctuate around zero. The original tangential pressure data in the ice phase are noisy due to the large fluctuation in density. To smooth the data, we apply the same one-dimensional Gaussian filter as in the density case. We also fit the raw data with a skewed Gaussian function (Supporting Information). In this planar interface, we distinguish the interfacial stress from the spherical nucleus one with the symbol *f*_|_, defined as^66^

$$f_{|}=\frac{1}{2}\int_{0}^{L_{y}}\;dy\left[P_{N}\left(y\right)-P_{T}\left(y\right)\right]$$ 4

where *L*_*y*_ is the length of the simulation box in the direction perpendicular to the interface (*y*) and the division by 2 accounts for the two interfaces present in the system due to periodic boundary conditions. We note that, while *P*_T_(*y*) is dependent on the contour, the integral over the entire system (and thus *f*_|_) in eq 4 is unique and free from the ambiguity of the contour definitions.^16,67^ After applying eq 4 to our trajectory, we obtain *f*_|_ ∼ 50 mJ/m^2^, almost twice the value of the interfacial free energy for the basal plane (γ_|_).^41,63^ This shows that the interfacial stress and interfacial free energy may differ in the ice–water interface. The interfacial stress seems notably more sensitive to thermodynamic changes than the interfacial free energy as previously observed using the mW model in ref 33. Last but not least, note that in the nucleus and the basal plane the interfacial stress is positive, as the interface is stretched to negative pressures.

**Figure 4:**
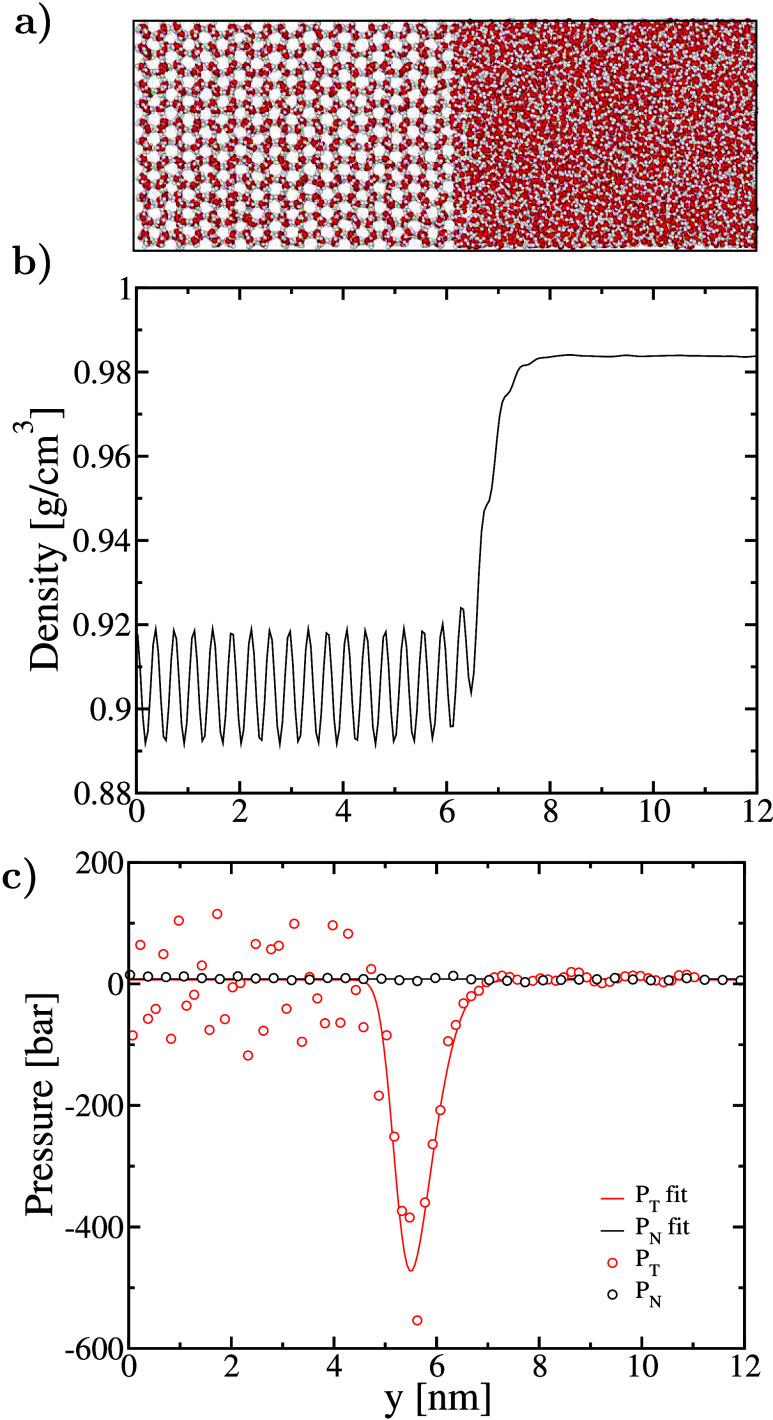
(a) Initial configuration of the system exposing the basal plane to the liquid and the secondary prismatic face to the reader. (b) Density and (c) pressure profiles across the ice–water interface exposing the basal plane at equilibrium at 270 K and 1 bar, obtained in the *Np*_*y*_ *T* ensemble. The density and tangential pressure points shown in the figure were those after smoothing using a one-dimensional Gaussian filter with σ = 3 Å.^64^ The tangential pressure fit (line) was generated by fitting the original data to a skewed Gaussian function. Panels b and c share the same horizontal axis, corresponding to the position along the direction normal to the interface (*y*-axis in the simulation box and analysis code). Raw data for density and pressure profiles are provided in the Supporting Information.

In summary, we have investigated the pressure inside a critical ice nucleus in supercooled water at 1 bar and 23 K of supercooling simulated via the TIP4P/Ice model. The planar interface for the basal plane is also studied to include a reference. The true (mechanical) pressure inside the ice nucleus is compared to the bulk ice reference (thermodynamic) pressure. The interfacial stress and free energies are examined in both systems. Our findings contrast notably with those of previous studies on hard-sphere and Lennard-Jones systems. While these simpler systems showed discrepancies between mechanical and thermodynamic approaches, our results show agreement for ice nucleation under the studied conditions.

However, we argue that this agreement may be just coincidental, as the interfacial stress was observed to be very sensitive to changes in conditions, as evidenced by our examination of the basal planar interface at a temperature of 270 K (i.e., the melting point of the model). There, the interfacial stress becomes approximately twice the magnitude of the interfacial free energy. Further work is needed to thoroughly elucidate the relation between *f* and γ during nucleation. What we have described here at 247 K and 1 bar may differ from what occurs at other temperatures and pressures. To rationalize the complex behavior of the interfacial stress, several factors may play a role, including interfacial mobility,^40^ density differences between the phases,^39^ bond energies,^39^ crystal strain, defects (e.g., vacancies),^6,7,13,68^ and anisotropy. Additional contributing factors may also be relevant.

The marked sensitivity to temperature changes of *f* strongly suggests that one should not rely upon mechanical routes for calculating γ, as they may lead to significant inaccuracies even under modest variations in conditions. Indeed, it is a conceptual error, as the interfacial free energy is defined from bulk reference states. In most cases, the liquid can be assumed to be bulk, and we have to find the reference bulk solid at the same chemical potential as the liquid. In confined systems,^18–26^ finding the reference bulk liquid may also be required.

We show that the pressure across an ice nucleus in supercooled water at 247 K may vary from an increased pressure of 200 bar in the core of the nucleus to a negative pressure at the interface before reaching the external pressure of 1 bar. Negative transversal pressures are also present in the basal plane. Our work provides insights into the relationship between mechanical and thermodynamic properties during ice nucleation, while also highlighting the limitations of mechanical approaches for interfacial free energy calculations. We hope to motivate further work along the line of harmonizing the thermodynamic and mechanical picture of solid–liquid interfaces.

## Supplementary Material



## Data Availability

The data to support these findings are available upon request.

## Notes

(1) Derivation of the pressure tensor in spherical coordinates, (2) governing equations for the local pressure tensor at a planar interface, (3) a complete description of the pressure tensor evaluation workflow (long-range corrections, fitting, and filtering), (4) raw planar interface data (density and pressure profiles), and (5) a discussion of the connection between thermodynamic and mechanical definitions of nucleus interfacial stress, with explicit consideration of the arbitrariness in defining the dividing surface and its location (PDF)
