# Supplementary material for: Comparing the Mechanical and Thermodynamic Definitions of Pressure in Ice Nucleation
Source: J Phys Chem Lett. 2026 Feb 12;17(8):2367–73. doi: 10.1021/acs.jpclett.5c03700 (PMC12951574; doi:10.1021/acs.jpclett.5c03700)
Supplement: Supplementary file 1 [file jz5c03700_si_001.pdf]

Supplementary Information: Comparing the Mechanical and Thermodynamic Definitions of Pressure in Ice Nucleation

P. Montero de Hijes[1], K. Shi[2], C. Vega[3], and C. Dellago[1]

January 28, 2026

[1]University of Vienna, Faculty of Physics, Kolingasse 14, A-1090 Vienna, Austria
[2]Department of Chemical and Biological Engineering, University at Buffalo, The State University of New York, Buffalo, NY 14260, USA
[3]Departamento de Química Física, Facultad de Ciencias Químicas, Universidad Complutense de Madrid, 28040 Madrid, Spain

kaihangs@buffalo.edu
christoph.dellago@univie.ac.at

In what follows, we show the derivation of molecular pressure tensor equations and calculation details.

1 Derivation of pressure tensor in spherical coordinates

Here we will present a full derivation for three diagonal elements in the local spherical pressure tensor for rigid molecules. A coarse-grained version has been presented in Ref. [1]. The derivation presented here follows the one for the hard sphere [2] but now we extend the formulation to rigid molecules.

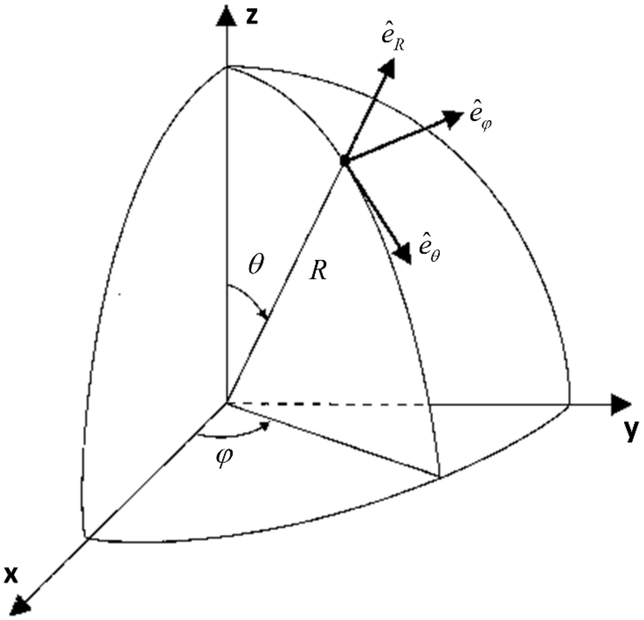

Figure 1: Spherical coordinate system with corresponding unit vectors in each direction. Adapted from Ref. [2]

In spherical coordinates, (R, θ, φ), due to the symmetry of the droplet, the local pressure tensor can be written as

$$\mathbf{P}(R) = P_N(R)\,\hat{e}_R\hat{e}_R + P_T(R)\left(\hat{e}_\theta\hat{e}_\theta + \hat{e}_\varphi\hat{e}_\varphi\right), \tag{1}$$

where $P_N = P_{RR}$ is the normal pressure in the radial direction; $P_T = P_{\theta\theta} = P_{\varphi\varphi}$ is the tangential pressure, and the polar component, $P_{\theta\theta}$, is equivalent to the azimuthal component, $P_{\varphi\varphi}$. The orthogonal unit vectors are

$$\hat{e}_R = \begin{pmatrix} \sin\theta\cos\varphi \\ \sin\theta\sin\varphi \\ \cos\theta \end{pmatrix} \hat{e}_\theta = \begin{pmatrix} \cos\theta\cos\varphi \\ \cos\theta\sin\varphi \\ -\sin\theta \end{pmatrix} \hat{e}_\varphi = \begin{pmatrix} -\sin\varphi \\ \cos\varphi \\ 0 \end{pmatrix}. \tag{2}$$

If a system is under the condition of hydrostatic equilibrium and there is no external field, we have $\nabla \cdot \mathbf{P} = 0$, i.e.,

$$P_T(R) = P_N(R) + \frac{R}{2}\frac{dP_N(R)}{dR}. \tag{3}$$

The total pressure tensor is composed of two parts: the kinetic part and the configurational part. The kinetic part is well defined and is given by $\mathbf{P}^K(R) = \rho(R)\,k_B T \mathbf{1}$, where $\rho(R)$ is the number density at a radial position R; k_B is the Boltzmann constant, T is temperature and $\mathbf{1}$ is the second order unit tensor. For molecules interacting in pairwise fashion, the configurational part is given by [3, 4]

$$\mathbf{P}^C(\mathbf{r}) = \frac{1}{2}\left\langle \sum_{i\neq j}\sum_a\sum_b \mathbf{F}_{iajb}\int_{C_{ij}} d\tilde{l}\,\delta\left(\mathbf{r}-\tilde{l}\right)\right\rangle, \tag{4}$$

where $\langle ... \rangle$ is the ensemble average; $\mathbf{F}_{iajb}$ is the force vector between interacting site a in molecule i and interacting site b in molecule j. Contour C_{ij} is defined as an arbitrary contour connecting the center of mass (COM) of two interacting molecules. Prefactor 1/2 is to account for double counting of molecules. The contour definition in the path integral was chosen to be that of Irving and Kirkwood [5, 6], which is a straight line connecting two interacting molecules:

$$\tilde{l} = \mathbf{r}_i + \alpha\mathbf{r}_{ij}, \tag{5}$$

where position vector $\mathbf{r}_{ij} = \mathbf{r}_j - \mathbf{r}_i$. Parameters α varies from 0 to 1. The derivative of the contour vector $\tilde{l}$ gives,

$$d\tilde{l} = \mathbf{r}_{ij}\,d\alpha. \tag{6}$$

The delta function in Eq. 4 can be rewritten in spherical coordinates as

$$\delta\left(\mathbf{r}-\tilde{l}\right) = \frac{1}{R^2\sin\theta}\delta(R-R_l)\,\delta(\theta-\theta_l)\,\delta(\varphi-\varphi_l). \tag{7}$$

Substituting Eq. 7 into Eq. 4 gives

$$\mathbf{P}^C(\mathbf{r}) = \frac{1}{2}\left\langle \sum_{i\neq j}\sum_a\sum_b \frac{\mathbf{r}_{iajb}}{r_{iajb}}F_{iajb}\frac{1}{R^2\sin\theta}\int_{C_{ij}} d\tilde{l}\,\delta(R-R_l)\,\delta(\theta-\theta_l)\,\delta(\varphi-\varphi_l)\right\rangle, \tag{8}$$

where r_{iajb} is the scalar distance and F_{iajb} is the scalar pair force.

1.1 Normal (Radial) Pressure P_N

The normal pressure is only a function of the radial distance R, we need to perform an average of the normal pressure over both polar and azimuthal angles so as to integrate out unnecessary variables (θ, φ),

$$P_N^C(R) = \frac{1}{4\pi}\int_0^{2\pi} d\varphi \int_0^{\pi} \sin\theta\, d\theta \left[\hat{e}_R \cdot \mathbf{P}^C(\mathbf{r}) \cdot \hat{e}_R\right]. \tag{9}$$

The bracket double dot product term in Eq. 9 essentially takes the diagonal element P_{RR}^C out of the 2nd-order pressure tensor. Substituting Eq. 8 into Eq. 9 gives,

$$P_N^C(R) = \frac{1}{8\pi R^2}\left\langle \sum_{i\neq j}\sum_a\sum_b \int_0^{2\pi} d\varphi\int_0^{\pi} d\theta \int_{C_{ij}} d\tilde{l}\cdot\hat{e}_R\frac{\mathbf{r}_{iajb}\cdot\hat{e}_R}{r_{iajb}}F_{iajb}\delta(R-R_l)\,\delta(\theta-\theta_l)\,\delta(\varphi-\varphi_l)\right\rangle. \tag{10}$$

Since

$$\int_{a-\varepsilon}^{a+\varepsilon} f\left(\alpha\right)\delta\left(\alpha-a\right)d\alpha = f\left(a\right), \varepsilon > 0, \tag{11}$$

we can perform the integration over polar and azimuthal angles and Eq. 10 becomes

$$P_N^C\left(R\right) = \frac{1}{8\pi R^2}\left\langle \sum_{i\neq j}\sum_a\sum_b \int_{C_{ij}} d\tilde{l}\cdot\hat{e}_{R,l}\frac{\mathbf{r}_{iajb}\cdot\hat{e}_{R,l}}{r_{iajb}}F_{iajb}\delta\left(R-R_l\right)\right\rangle, \tag{12}$$

where $\hat{e}_{R,l}$ is the radial unit vector with $\theta=\theta_l$ and $\varphi=\varphi_l$. Using the differential in Eq. 6 and using the identity,

$$\int_c^d f\left(\alpha\right)d\alpha = \int_{-\infty}^{+\infty} f\left(\alpha\right)H\left(\alpha-c\right)H\left(d-\alpha\right)d\alpha. \tag{13}$$

Eq. 12 becomes

$$\begin{aligned} P_N^C\left(R\right) &= \frac{1}{8\pi R^2}\left\langle \sum_{i\neq j}\sum_a\sum_b \int_0^1 d\alpha\frac{\left(\mathbf{r}_{ij}\cdot\hat{e}_{R,l}\right)\left(\mathbf{r}_{iajb}\cdot\hat{e}_{R,l}\right)}{r_{iajb}}F_{iajb}\delta\left(R-R_l\right)\right\rangle \\ &= \frac{1}{8\pi R^2}\left\langle \sum_{i\neq j}\sum_a\sum_b \int_{-\infty}^{+\infty} d\alpha\frac{\left(\mathbf{r}_{ij}\cdot\hat{e}_{R,l}\right)\left(\mathbf{r}_{iajb}\cdot\hat{e}_{R,l}\right)}{r_{iajb}}F_{iajb}H\left(\alpha\right)H\left(1-\alpha\right)\delta\left(R-R_l\right)\right\rangle \end{aligned} \tag{14}$$

where $H\left(x\right)$ is the Heaviside step function. Applying Eq. 11 and the identity

$$\delta\left[f\left(\alpha\right)\right] = \sum_k \frac{1}{\left|f'\left(\alpha_k\right)\right|}\delta\left(\alpha-\alpha_k\right), \tag{15}$$

where $f'\left(\alpha\right)=df/d\alpha$ and α_k is a simple root of $f\left(\alpha_k\right)=0$, to Eq. 14 yields

$$\begin{aligned} P_N^C\left(R\right) &= \frac{1}{8\pi R^2}\left\langle \sum_{i\neq j}\sum_a\sum_b \int_{-\infty}^{+\infty} d\alpha\frac{\left(\mathbf{r}_{ij}\cdot\hat{e}_{R,l}\right)\left(\mathbf{r}_{iajb}\cdot\hat{e}_{R,l}\right)}{r_{iajb}}F_{iajb}H\left(\alpha\right)H\left(1-\alpha\right)\sum_k\frac{1}{\left|\mathbf{r}_{ij}\cdot\hat{e}_{R,l}\right|}\delta\left(\alpha-\alpha_k\right)\right\rangle \\ &= \frac{1}{8\pi R^2}\left\langle \sum_{i\neq j}\sum_a\sum_b\sum_k \frac{\left(\mathbf{r}_{ij}\cdot\hat{e}_{R,\alpha_k}\right)\left(\mathbf{r}_{iajb}\cdot\hat{e}_{R,\alpha_k}\right)}{\left|\mathbf{r}_{ij}\cdot\hat{e}_{R,\alpha_k}\right|}\frac{F_{iajb}}{r_{iajb}}H\left(\alpha_k\right)H\left(1-\alpha_k\right)\right\rangle, \end{aligned} \tag{16}$$

recalling $R_l\left(\alpha\right)=\sqrt{\left(x_i+\alpha x_{ij}\right)^2+\left(y_i+\alpha y_{ij}\right)^2+\left(z_i+\alpha z_{ij}\right)^2}$ and α_k are the roots of a quadratic equation, $R=R_l$, i.e.,

$$\left(\mathbf{r}_{ij}\right)^2\alpha^2+2\alpha\mathbf{r}_i\cdot\mathbf{r}_{ij}+\left(\mathbf{r}_i\right)^2-R^2=0. \tag{17}$$

If the discriminant of Eq. 17 is smaller than zero, there is no contribution to the surface R; if the discriminant is equal to zero, contour $\tilde{l}$ is tangent to the spherical surface of radius R, and $\mathbf{r}_{ij}\cdot\hat{e}_{R,\alpha_k}=0$ thus there is no contribution to the surface R either. Only if the discriminant is larger than zero, the force between ij-molecule then contributes to the local pressure (but α_k must be between 0 and 1). The radial unit vector $\hat{e}_{R,\alpha_k}$ at $\alpha=\alpha_k$ is given by

$$\hat{e}_{R,\alpha_k} = \begin{pmatrix} (x_i+\alpha_k x_{ij})/R \\ (y_i+\alpha_k y_{ij})/R \\ (z_i+\alpha_k z_{ij})/R \end{pmatrix}. \tag{18}$$

Because the contour from i to j and the contour from j to i are physically equivalent, we can further simplify Eq. 16 and do the summation over pairs ($i<j$),

$$P_N^C\left(R\right) = \frac{1}{4\pi R^2}\left\langle \sum_{i<j}\sum_a\sum_b\sum_{k=1}^{2} \frac{\left(\mathbf{r}_{ij}\cdot\hat{e}_{R,\alpha_k}\right)\left(\mathbf{r}_{iajb}\cdot\hat{e}_{R,\alpha_k}\right)}{\left|\mathbf{r}_{ij}\cdot\hat{e}_{R,\alpha_k}\right|}\frac{F_{iajb}}{r_{iajb}}H\left(\alpha_k\right)H\left(1-\alpha_k\right)\right\rangle. \tag{19}$$

Combining the kinetic and configurational contributions together, the total normal pressure is

$$P_N(R) = \rho(R)\,k_BT + \frac{1}{4\pi R^2}\left\langle \sum_{i<j}\sum_a\sum_b\sum_{k=1}^{2} \frac{(\mathbf{r}_{ij}\cdot\hat{e}_{R,\alpha_k})(\mathbf{r}_{iajb}\cdot\hat{e}_{R,\alpha_k})}{|\mathbf{r}_{ij}\cdot\hat{e}_{R,\alpha_k}|}\frac{F_{iajb}}{r_{iajb}} H(\alpha_k)\,H(1-\alpha_k)\right\rangle. \quad (20)$$

1.2 Polar Pressure $P_{\theta\theta}$

The local polar pressure only depends on the radial distance R, an integration over the polar and azimuthal angles must be carried out in a similar manner to Eq. 9. The polar pressure component is given by

$$P^C_{\theta\theta}(R) = \frac{1}{4\pi}\int_0^{2\pi} d\varphi \int_0^{\pi} \sin\theta d\theta \left[\hat{e}_\theta \cdot \mathbf{P}^C(\mathbf{r}) \cdot \hat{e}_\theta\right]. \quad (21)$$

Applying Eq. 8 gives

$$P^C_{\theta\theta}(R) = \frac{1}{8\pi R^2}\left\langle \sum_{i\neq j}\sum_a\sum_b \int_0^{2\pi} d\varphi \int_0^{\pi} d\theta \int_{C_{ij}} d\tilde{l}\cdot\hat{e}_\theta \frac{\mathbf{r}_{iajb}\cdot\hat{e}_\theta}{r_{iajb}} F_{iajb}\delta(R-R_l)\,\delta(\theta-\theta_l)\,\delta(\varphi-\varphi_l)\right\rangle. \quad (22)$$

Integrating out variable θ and φ leads to

$$P^C_{\theta\theta}(R) = \frac{1}{8\pi R^2}\left\langle \sum_{i\neq j}\sum_a\sum_b \int_{C_{ij}} d\tilde{l}\cdot\hat{e}_{\theta,l} \frac{\mathbf{r}_{iajb}\cdot\hat{e}_{\theta,l}}{r_{iajb}} F_{iajb}\delta(R-R_l)\right\rangle, \quad (23)$$

where $\hat{e}_{\theta,l}$ is the polar unit vector with $\theta = \theta_l$ and $\varphi = \varphi_l$. Now, replacing the line element $d\tilde{l}$ in Eq. 23 with Eq. 6 results in

$$P^C_{\theta\theta}(R) = \frac{1}{8\pi R^2}\left\langle \sum_{i\neq j}\sum_a\sum_b \int_0^1 d\alpha \frac{(\mathbf{r}_{ij}\cdot\hat{e}_{\theta,l})(\mathbf{r}_{iajb}\cdot\hat{e}_{\theta,l})}{r_{iajb}} F_{iajb}\delta(R-R_l)\right\rangle. \quad (24)$$

Applying the identity (Eq. 15) to above Eq. 24 and carrying out the integration yields,

$$P^C_{\theta\theta}(R) = \frac{1}{8\pi R^2}\left\langle \sum_{i\neq j}\sum_a\sum_b\sum_{k=1}^{2} \frac{(\mathbf{r}_{ij}\cdot\hat{e}_{\theta,\alpha_k})(\mathbf{r}_{iajb}\cdot\hat{e}_{\theta,\alpha_k})}{|\mathbf{r}_{ij}\cdot\hat{e}_{R,\alpha_k}|}\frac{F_{iajb}}{r_{iajb}} H(\alpha_k)\,H(1-\alpha_k)\right\rangle, \quad (25)$$

where

$$\hat{e}_{\theta,\alpha_k} = \begin{pmatrix} (x_i+\alpha_k x_{ij})(z_i+\alpha_k z_{ij})/(Rd_{xy}) \\ (y_i+\alpha_k y_{ij})(z_i+\alpha_k z_{ij})/(Rd_{xy}) \\ -d_{xy}/R \end{pmatrix}, \quad (26)$$

and $d_{xy} = \sqrt{(x_i+\alpha_k x_{ij})^2 + (y_i+\alpha_k y_{ij})^2}$; α_k are roots of Eq. 17. Thus, the local polar pressure is given by

$$P_{\theta\theta}(R) = \rho(R)\,k_BT + \frac{1}{4\pi R^2}\left\langle \sum_{i<j}\sum_a\sum_b\sum_{k=1}^{2} \frac{(\mathbf{r}_{ij}\cdot\hat{e}_{\theta,\alpha_k})(\mathbf{r}_{iajb}\cdot\hat{e}_{\theta,\alpha_k})}{|\mathbf{r}_{ij}\cdot\hat{e}_{R,\alpha_k}|}\frac{F_{iajb}}{r_{iajb}} H(\alpha_k)\,H(1-\alpha_k)\right\rangle. \quad (27)$$

1.3 Azimuthal Pressure $P_{\varphi\varphi}$

Following the similar derivation as shown above, we can obtain the local azimuthal pressure in a form of

$$P_{\varphi\varphi}(R) = \rho(R)\,k_BT + \frac{1}{4\pi R^2}\left\langle \sum_{i<j}\sum_a\sum_b\sum_{k=1}^{2} \frac{(\mathbf{r}_{ij}\cdot\hat{e}_{\varphi,\alpha_k})(\mathbf{r}_{iajb}\cdot\hat{e}_{\varphi,\alpha_k})}{|\mathbf{r}_{ij}\cdot\hat{e}_{R,\alpha_k}|}\frac{F_{iajb}}{r_{iajb}} H(\alpha_k)\,H(1-\alpha_k)\right\rangle, \quad (28)$$

where

$$\hat{e}_{\varphi,\alpha_k} = \begin{pmatrix} -(y_i+\alpha_k y_{ij})/d_{xy} \\ (x_i+\alpha_k x_{ij})/d_{xy} \\ 0 \end{pmatrix}. \quad (29)$$

The final tangential pressure is calculated by $P_T(R) = [P_{\theta\theta}(R) + P_{\varphi\varphi}(R)]/2$.

2 Local pressure tensor equations for the planar interface

For systems with y-axis being vertical to the plane, and x/z being tangential. Diagonal elements in the pressure tensor can be calculated [7] using

$$P_{xx}(y) = \frac{1}{S}\left\langle \sum_{i<j}\sum_{a}\sum_{b} \frac{x_{iajb}x_{ij}}{r_{iajb}|y_{ij}|} F_{iajb} H\left(\frac{y-y_i}{y_{ij}}\right) H\left(\frac{y_j-y}{y_{ij}}\right)\right\rangle \tag{30}$$

$$P_{zz}(y) = \frac{1}{S}\left\langle \sum_{i<j}\sum_{a}\sum_{b} \frac{z_{iajb}z_{ij}}{r_{iajb}|y_{ij}|} F_{iajb} H\left(\frac{y-y_i}{y_{ij}}\right) H\left(\frac{y_j-y}{y_{ij}}\right)\right\rangle \tag{31}$$

$$P_{yy}(y) = \frac{1}{S}\left\langle \sum_{i<j}\sum_{a}\sum_{b} \frac{y_{iajb}y_{ij}}{r_{iajb}|y_{ij}|} F_{iajb} H\left(\frac{y-y_i}{y_{ij}}\right) H\left(\frac{y_j-y}{y_{ij}}\right)\right\rangle \tag{32}$$

Where S is the surface area of the planar interface, subscript $iajb$ indicates the atom a in molecule i and atom b in molecule j. $H(x)$ is the Heaviside step function. The normal pressure is $P_N = P_{yy}$ and the tangential pressure is calculated by averaging over both x- and z-directions, i.e., $P_T = 1/2(P_{xx} + P_{zz})$. Off-diagonal elements in the pressure tensor represent the shear stress of the system and they can be calculated by:

$$P_{xy}(y) = \frac{1}{S}\left\langle \sum_{i<j}\sum_{a}\sum_{b} \frac{x_{iajb}y_{ij}}{r_{iajb}|y_{ij}|} F_{iajb} H\left(\frac{y-y_i}{y_{ij}}\right) H\left(\frac{y_j-y}{y_{ij}}\right)\right\rangle \tag{33}$$

$$P_{xz}(y) = \frac{1}{S}\left\langle \sum_{i<j}\sum_{a}\sum_{b} \frac{x_{iajb}z_{ij}}{r_{iajb}|y_{ij}|} F_{iajb} H\left(\frac{y-y_i}{y_{ij}}\right) H\left(\frac{y_j-y}{y_{ij}}\right)\right\rangle \tag{34}$$

$$P_{yx}(y) = \frac{1}{S}\left\langle \sum_{i<j}\sum_{a}\sum_{b} \frac{y_{iajb}x_{ij}}{r_{iajb}|y_{ij}|} F_{iajb} H\left(\frac{y-y_i}{y_{ij}}\right) H\left(\frac{y_j-y}{y_{ij}}\right)\right\rangle \tag{35}$$

$$P_{yz}(y) = \frac{1}{S}\left\langle \sum_{i<j}\sum_{a}\sum_{b} \frac{y_{iajb}z_{ij}}{r_{iajb}|y_{ij}|} F_{iajb} H\left(\frac{y-y_i}{y_{ij}}\right) H\left(\frac{y_j-y}{y_{ij}}\right)\right\rangle \tag{36}$$

$$P_{zx}(y) = \frac{1}{S}\left\langle \sum_{i<j}\sum_{a}\sum_{b} \frac{z_{iajb}x_{ij}}{r_{iajb}|y_{ij}|} F_{iajb} H\left(\frac{y-y_i}{y_{ij}}\right) H\left(\frac{y_j-y}{y_{ij}}\right)\right\rangle \tag{37}$$

$$P_{zy}(y) = \frac{1}{S}\left\langle \sum_{i<j}\sum_{a}\sum_{b} \frac{z_{iajb}y_{ij}}{r_{iajb}|y_{ij}|} F_{iajb} H\left(\frac{y-y_i}{y_{ij}}\right) H\left(\frac{y_j-y}{y_{ij}}\right)\right\rangle \tag{38}$$

3 Pressure tensor calculation details

3.1 Long-range corrections

The water model is taken to be TIP4P/Ice [8]. All parameters in pressure tensor calculations are consistent with those in the molecular dynamics simulations, except for the long-range interactions. We adopted the shifted-force version [9, 10] of the Wolf method [11] to account for the Coulombic interactions in pressure tensor calculations, and the Coulombic force was calculated by

$$F_{coul}\left(r_{iajb}\right) = q_{ia}q_{jb}\left[\left(\frac{erfc(\alpha r_{iajb})}{{r_{iajb}}^2} + \frac{2\alpha}{\sqrt{\pi}}\frac{e^{-\alpha^2 {r_{iajb}}^2}}{r_{iajb}}\right) - \left(\frac{erfc(\alpha r_c)}{{r_c}^2} + \frac{2\alpha}{\sqrt{\pi}}\frac{e^{-\alpha^2 {r_c}^2}}{r_c}\right)\right], \tag{39}$$

where r_c is the cutoff radius for Coulombic interactions. For pressure tensor calculations of ice nucleus, r_c was taken to be 65 Angstrom (half of the simulation box size). Mendoza et al. [12] reported that the shifted-force version gives poor surface tension results compared to the Ewald summation method, but they used a small cutoff radius which might be the source of error (and this has been confirmed in Ref. [13]). It has been shown that increasing cutoff radius in real space, the Fourier space contribution to the pressure becomes smaller, and the systematic error introduced by replacing the bare Coulombic potential with a damped one becomes negligible [4]. Potential damping

parameter α in Eq. 39 was chosen by running pressure calculations at different values (from 0.005 to 0.3), and we found a plateau in $\alpha - P_{N,ext}$ plot in the α range from 0.05 to 0.2, where $P_{N,ext}$ is the normal pressure in the external liquid water. We chose $\alpha = 0.06$ to give the best match between averaged pressure (from MD simulation) and the local pressure in the external liquid water phase. The force equation (shifted-force version, Eq. 39) is the same as that in the original Wolf potential, but in the original version, the force and potential are not consistent, while in shifted-force version, this problem has been fixed. For pressure tensor calculations of planar systems, we found that $r_c = 20$ Angstrom and $\alpha = 0.195$ are enough to capture long-range information within uncertainty introduced by the Wolf method. However, using a smaller cutoff radius reduces the computational cost significantly. The periodic boundary conditions were implemented in the pressure tensor calculations for both spherical and planar cases, using the algorithm of Ref. [4]. The source code for spherical and planar local pressure tensor calculations is available at `https://github.com/KaihangShi/Spherical-Pressure-Tensor`.

3.2 Fitting of the pressure components in spherical coordinates

Since the data for the transversal component P_T was rather noisy, instead of directly fitting it, we obtained its profile from the fit to the normal component and the mechanical equilibrium condition, which implies Eq. SI.3. The discrete P_N data were fitted to a hyperbolic equation [6, 14],

$$P_N(R) = \frac{1}{2}(P_{I_h} + P_w) + \frac{P_w}{2}\tanh\left[\frac{2(R - R_w)}{D_w}\right] - \frac{P_{I_h}}{2}\tanh\left[\frac{2(R - R_{I_h})}{D_{I_h}}\right], \tag{40}$$

where subscript 'I_h' denotes ice and 'w' water; P_{I_h} and P_w are the normal pressure of the bulk solid and that of the bulk liquid, respectively; R_{I_h} and R_w are the radial positions of the inflection points on the normal pressure profile for the corresponding phase; D_{I_h} and D_w are the width of the interface for the corresponding phase. Fitting parameters are listed in Table 1.

**Systems**	P_w (bar)	P_{I_h} (bar)	R_w (Å)	R_{I_h} (Å)	D_w (Å)	D_{I_h} (Å)
Ice nucleus	10.2	188.8	12.7	24.3	5.0	9.9

Table 1: Fit parameters for Ice Nucleus System

3.3 Smoothing method for tangential pressure profile in planar interfaces using the skewed Gaussian function

In systems with planar interfaces, the normal pressure in the direction perpendicular to the interface is constant according to the hydrostatic equilibrium (see Figure 4). The tangential pressure profile in a solid-liquid interface is well-known to be noisy due to the high short-range order of the crystal structure. To obtain a smooth tangential pressure that is amenable to numerical analysis, we fit the noisy tangential pressure profile using a skewed Gaussian function (assuming y-axis is vertical to the planar interface):

$$P_T(y) = A\left[1 + \operatorname{erf}\left(\frac{\alpha(y - y_0)}{\sqrt{2}}\right)\right]\exp\left(-\frac{(y - y_0)^2}{2\sigma^2}\right) + P \tag{41}$$

Where A, α, y_0, σ are fitting parameters. P is the mechanical pressure in the bulk liquid/solid phase of the system and it is equal to the normal pressure; in this case, $P = 6.686$ bar. To enhance the fitting quality, we first numerically integrated the raw tangential pressure data from the liquid phase to the solid phase using the trapezoidal rule (referred to as "raw integral"). Then we fit the raw integral with the integral of the skewed Gaussian function to obtain the fitting parameters A, α, y_0, σ. The fitting parameters are stable with various initial guesses. A Jupyter notebook to perform the smoothing of the original tangential pressure data points is available at: `https://github.com/KaihangShi/Spherical-Pressure-Tensor/tree/main/TIP4P_Ice`

4 Raw data for the basal plane

Here the data without post-processing is shown for the planar interface exposing the basal facet.

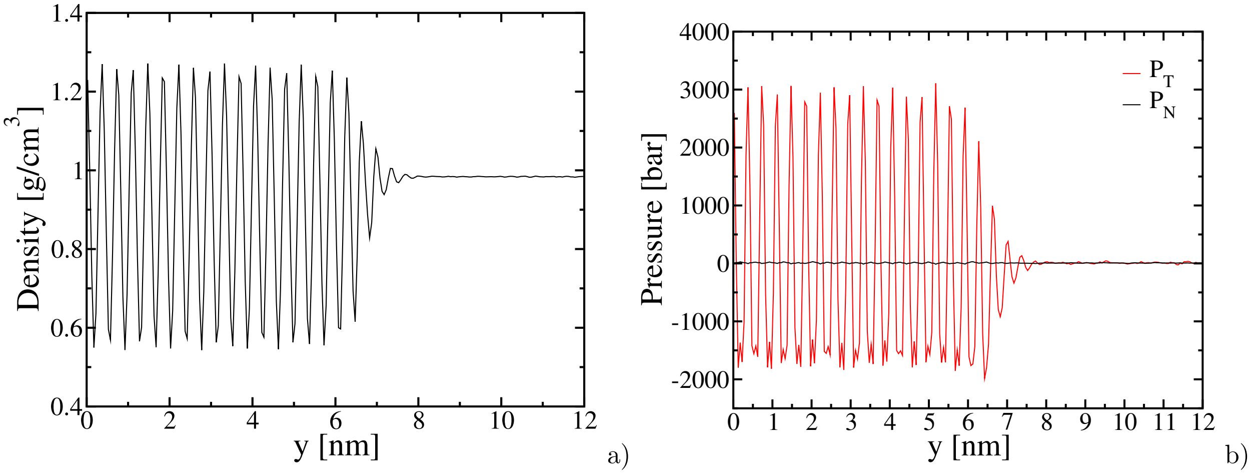

Figure 2: a) Density and b) pressure profiles across the ice-water interface exposing the basal plane without smoothing of data.

5 Connecting the thermodynamic and mechanical definitions of the interfacial stress of the nucleus

Following Eq.(14) from Ref. [6], we see the following mechanical definition for the interfacial stress f,

$$f = \int_0^\infty \frac{R^*}{r}[P_N(r) - P_T(r)]dr, \tag{42}$$

where R^* denotes a particular location of the dividing surface, which, in principle, depends on the specific definition of the pressure tensor. Note that $P_N(r) - P_T(r)$ is nonzero only within the interfacial region. Consequently, the integrand differs from zero only in this region, where r approaches R^*, i.e., $R^*/r \sim 1$. Under this assumption, Eq. (42) can be approximated, leading to Eq. (4) of the main text for $f_|$ (up to a factor of 2 accounting for the presence of two interfaces due to periodic boundary conditions). Indeed, Ref. [15] showed that employing a planar-interface expression together with the pressure profiles of the nucleus yields an estimate of f comparable to that obtained from a rigorous spherical-interface formulation. The mechanical equilibrium condition in spherical coordinates is

$$P_T(r) = P_N(r) + \frac{r}{2}\frac{dP_N(r)}{dr}, \tag{43}$$

which can be re-arranged as

$$\frac{P_N(r) - P_T(r)}{r} = -\frac{1}{2}\frac{dP_N(r)}{dr}. \tag{44}$$

Substituting Eq. 44 into Eq. 42, we get:

$$f = \int_0^\infty -\frac{R^*}{2}\frac{dP_N(r)}{dr}dr \quad = -\frac{R^*}{2}\int_0^\infty \left[\frac{dP_N(r)}{dr}\right]dr \ = -\frac{R^*}{2}P_N(r)\Big|_{r=0}^{r=\infty} \quad = -\frac{R^*}{2}(p_w - p_{I_h}) \ = \frac{R^*}{2}(p_{Ih} - p_w),$$

with $p_{Ih} = P_N(r \sim 0)$ and $p_w = P_N(r \sim \infty)$.

Let's find the same equation for f but using thermodynamic arguments. We follow the book of Rowlinson and Widom [16] adapting the equations to deal with the true properties of the nucleus and the interfacial stress f, instead of bulk properties and the interfacial free energy γ. Hence

$$F = -p_w V_w - p_{I_h} V_{I_h} + fA + \mu N, \tag{45}$$

where F means Helmholtz free energy, p_w and p_{I_h}, and V_w and V_{I_h}, are the pressures and volumes of liquid and ice respectively (note that the dividing surface has no volume), μ is the chemical potential (which is homogeneous in equilibrium), and $N = N_w + N_{I_h} + N_x$ is the total number of molecules (liquid w, ice I_h, and excess components x). By considering a change in the location of the dividing surface (denoted in brackets) under which free energies, pressure, and chemical potential do not change, we find,

$$[dF] = -p_w[dV_w] - p_{I_h}[dV_{I_h}] + f[dA] + A\left[\frac{df}{dR}\right][dR] = 0, \tag{46}$$

which can be re-arranged to give

$$p_{I_h} - p_w = \frac{2f}{R} + \left[\frac{df}{dR}\right]. \tag{47}$$

Now, it is possible to define a particular choice of R that cancels the notational derivative, namely R^*. This can be called the *true* surface of tension, which may differ from the surface of tension R_s defined by zeroing the notational derivative of γ. As can be seen, setting R to R^*, we recover the same equation as from mechanical arguments,

$$f = \frac{R^*}{2}(p_{I_h} - p_w) \tag{48}$$

We acknowledge that further work is needed to connect the surface of tension R_s and the true surface of tension R^* since they are not necessarily equal as shown in Ref. [17]. Moreover, R^* obtained from mechanical arguments depends on the choice of the pressure tensor [6]. An attempt to connect R_s and R^* follows hereafter assuming that R^* is well-defined (at least that is the case in thermodynamics). By comparing, Eq. 47 with the general Young-Laplace equation,

$$p^{\mu}_{I_h} - p_w = \frac{2\gamma}{R} + \left[\frac{d\gamma}{dR}\right], \tag{49}$$

we find,

$$p^{\mu}_{I_h} - p_{I_h} = \frac{2}{R}(\gamma - f) + \left[\frac{d}{dR}\right](\gamma - f). \tag{50}$$

Let us define $\Delta p_{I_h} = p^{\mu}_{I_h} - p_{I_h}$ and $u = \gamma - f$ for notational simplicity. The equation becomes:

$$\Delta p_{I_h} = \frac{2u}{R} + \left[\frac{du}{dR}\right], \tag{51}$$

which is a first-order linear differential equation. To solve this equation, we employ the method of integrating factors. Let $\mu_0(R)$ be the integrating factor:

$$\mu_0(R) = \exp\left(\int -\frac{2}{R}dR\right) = \exp(-2\ln R) = \frac{1}{R^2}. \tag{52}$$

Re-arranging and multiplying both sides of Eq. 51 by $\mu_0(R)$, one obtains

$$\frac{1}{R^2}\left[\frac{du}{dR}\right] - \left(\frac{2}{R^3}\right)u = \frac{\Delta p_{I_h}}{R^2}, \tag{53}$$

or,

$$\left[\frac{d}{dR}\right]\left(\frac{u}{R^2}\right) = \frac{\Delta p_{I_h}}{R^2}, \tag{54}$$

which can be integrated to give,

$$\frac{u}{R^2} = -\frac{\Delta p_{I_h}}{R} + C, \tag{55}$$

where C is a constant of integration. To determine the constant C, we use the condition that at $R = R_s$, $\left[\frac{d\gamma}{dR}\right] = 0$. Taking the derivative in Eq. 55 substituting $u = \gamma - f$,

$$\left[\frac{d}{dR}\right](\gamma - f) = -\Delta p_{I_h} + 2CR, \tag{56}$$

where at $R = R_s$, we have:

$$\left[\frac{d}{dR}\right](\gamma - f) = \left[\frac{d\gamma}{dR}\right] - \left[\frac{df}{dR}\right] = -\left[\frac{df}{dR}\right]. \tag{57}$$

Therefore,

$$C = \frac{1}{2R_s}\left(\Delta p_{I_h} - \left[\frac{df}{dR}\right]_{R_s}\right), \tag{58}$$

and the difference between γ and f at any dividing surface R is given as

$$\gamma - f = \Delta p_{I_h} R\left(\frac{R}{2R_s} - 1\right) - \frac{R^2}{2R_s}\left[\frac{df}{dR}\right]_{R_s}. \tag{59}$$

We could have also used the condition that at $R = R^*$, $\left[\frac{df}{dR}\right] = 0$. In that case,

$$C = \frac{1}{2R^*}\left(\Delta p_{I_h} + \left[\frac{d\gamma}{dR}\right]_{R^*}\right), \tag{60}$$

and we find,

$$R^* = R_s\left(\frac{\Delta p_{I_h} + \left[\frac{d\gamma}{dR}\right]_{R^*}}{\Delta p_{I_h} - \left[\frac{df}{dR}\right]_{R_s}}\right), \tag{61}$$

which shows that the relation between the surface of tension R_s and the true surface of tension R^* is not simple. It is common to find in the literature that these two are not distinguished. However, this is an approximation. Since obtaining exactly these quantities requires several costly free energy calculations of F for the whole system, it may be more convenient from a practical point of view to use values of R covering the whole interfacial layer to estimate f and γ.

References

[1] S. M. A. Malek, F. Sciortino, P. H. Poole, and I. Saika-Voivod, "Evaluating the Laplace pressure of water nanodroplets from simulations," *Journal of Physics: Condensed Matter*, vol. 30, p. 144005, 4 2018.

[2] P. Montero de Hijes, K. Shi, E. G. Noya, E. E. Santiso, K. E. Gubbins, E. Sanz, and C. Vega, "The Young–Laplace equation for a solid–liquid interface," *The Journal of Chemical Physics*, vol. 153, p. 191102, 11 2020.

[3] P. Schofield and J. R. Henderson, "Statistical Mechanics of Inhomogeneous Fluids," *Proceedings of the Royal Society A: Mathematical, Physical and Engineering Sciences*, vol. 379, pp. 231–246, 1 1982.

[4] K. Shi, Y. Shen, E. E. Santiso, and K. E. Gubbins, "Microscopic Pressure Tensor in Cylindrical Geometry: Pressure of Water in a Carbon Nanotube," *Journal of Chemical Theory and Computation*, vol. 16, pp. 5548–5561, 9 2020.

[5] J. H. Irving and J. G. Kirkwood, "The Statistical Mechanical Theory of Transport Processes. IV. The Equations of Hydrodynamics," *The Journal of Chemical Physics*, vol. 18, pp. 817–829, 6 1950.

[6] S. M. Thompson, K. E. Gubbins, J. P. R. B. Walton, R. A. R. Chantry, and J. S. Rowlinson, "A molecular dynamics study of liquid drops," *The Journal of Chemical Physics*, vol. 81, pp. 530–542, 7 1984.

[7] J. Alejandre, D. J. Tildesley, and G. A. Chapela, "Molecular dynamics simulation of the orthobaric densities and surface tension of water," *The Journal of Chemical Physics*, vol. 102, pp. 4574–4583, 3 1995.

[8] J. L. F. Abascal, E. Sanz, R. García Fernández, and C. Vega, "A potential model for the study of ices and amorphous water: TIP4P/Ice," *The Journal of Chemical Physics*, vol. 122, p. 234511, 6 2005.

[9] D. Zahn, B. Schilling, and S. M. Kast, "Enhancement of the Wolf Damped Coulomb Potential: Static, Dynamic, and Dielectric Properties of Liquid Water from Molecular Simulation," *The Journal of Physical Chemistry B*, vol. 106, pp. 10725–10732, 10 2002.

[10] C. J. Fennell and J. D. Gezelter, "Is the Ewald summation still necessary? Pairwise alternatives to the accepted standard for long-range electrostatics," *The Journal of Chemical Physics*, vol. 124, p. 234104, 6 2006.

[11] D. Wolf, P. Keblinski, S. R. Phillpot, and J. Eggebrecht, "Exact method for the simulation of Coulombic systems by spherically truncated, pairwise r1 summation," *The Journal of Chemical Physics*, vol. 110, pp. 8254–8282, 5 1999.

[12] F. N. Mendoza, J. López-Lemus, G. A. Chapela, and J. Alejandre, "The Wolf method applied to the liquid-vapor interface of water," *The Journal of Chemical Physics*, vol. 129, p. 024706, 7 2008.

[13] K. Z. Takahashi, T. Narumi, and K. Yasuoka, "Cutoff radius effect of the isotropic periodic sum and Wolf method in liquid–vapor interfaces of water," *The Journal of Chemical Physics*, vol. 134, p. 174112, 5 2011.

[14] J. Vrabec, G. K. Kedia, G. Fuchs, and H. Hasse, "Comprehensive study of the vapour–liquid coexistence of the truncated and shifted Lennard–Jones fluid including planar and spherical interface properties," *Molecular Physics*, vol. 104, pp. 1509–1527, 5 2006.

[15] M. de Jager, C. Vega, P. Montero de Hijes, F. Smallenburg, and L. Filion, "Statistical mechanics of crystal nuclei of hard spheres," *The Journal of Chemical Physics*, vol. 161, no. 18, 2024.

[16] J. S. Rowlinson and B. Widom, *Molecular Theory of Capillarity.* Oxford: Clarendon Press, 1982.

[17] P. R. ten Wolde and D. Frenkel, "Computer simulation study of gas–liquid nucleation in a Lennard-Jones system," *The Journal of Chemical Physics*, vol. 109, pp. 9901–9918, 12 1998.
